# Impact of sleep apnea on alzheimer’s disease in relation to sex: an 8-year longitudinal follow-up study of a nationwide cohort

**DOI:** 10.1186/s13195-024-01667-6

**Published:** 2025-03-20

**Authors:** Su Jin Chung, Sung Hoon Kang, Minwoong Kang, Yunjin Choi, Yu Jeong Park, Hayom Kim, Kyungmi Oh, Seong-Beom Koh, Jung Bin Kim

**Affiliations:** 1 Department of Neurology, Inje University Ilsan Paik Hospital, Inje University College of Medicine, 170 Juhwa-ro, Ilsanseo-gu, Goyang-si, Gyeonggi-do 10380 South Korea; 2 Department of Neurology, Korea University Guro Hospital, Korea University College of Medicine, 148 Gurodong-ro, Guro-gu, Seoul, 08308 South Korea; 3 Department of Biomedical Research Center, Korea University Guro Hospital, Korea University College of Medicine, 148 Gurodong-ro, Guro-gu, Seoul, 08308 South Korea; 4 Department of Neurology, Korea University Anam Hospital, Korea University College of Medicine, 73 Goryeodae-ro, Seongbuk-gu, Seoul, 02841 South Korea

**Keywords:** Sleep apnea, Sex, Obesity, Dementia of the Alzheimer type (DAT)

## Abstract

**Background:**

We aimed to investigate the association between sleep apnea and incident dementia (dementia of the Alzheimer type [DAT] and vascular dementia) and whether differences in the effects of sleep apnea on dementia depend on sex. Furthermore, we sought to determine whether obesity affects the sex-specific relationship between sleep apnea and dementia.

**Methods:**

We used de-identified data on patients with sleep apnea and a control group aged ≥ 50 years from the Korean National Health Insurance Service. After propensity score matching to balance age and sex between the patient and control groups, 30,111 individuals with sleep apnea (patient group) and 121,528 individuals without sleep apnea (control group) were included. To investigate the impact of sleep apnea on the development of dementia, we used Cox proportional hazards regression after controlling for potential confounders.

**Results:**

Sleep apnea was predictive of developing DAT in both women (hazard ratio [HR] = 1.30, 95% confidence interval [CI] 1.16–1.44, *p* < 0.001) and men (HR = 1.13, 95% CI 1.03–1.24, *p* = 0.012). The adverse effects of sleep apnea on DAT were more prominent in women than in men (*p* = 0.015 for sleep apnea×sex). Furthermore, obesity affected the sex-specific relationship between sleep apnea and DAT. Specifically, the adverse effects of obese sleep apnea on the DAT were more pronounced in women than in men (*p* = 0.002 for obese sleep apnea×sex). In contrast, there were no differences in the effects of non-obese sleep apnea on DAT between women and men (*p* = 0.667 for non-obese sleep apnea×sex).

**Conclusions:**

Our results highlight sex differences in the adverse effects of sleep apnea on DAT. Furthermore, these results suggest that sex-specific strategies for controlling sleep apnea are necessary to prevent DAT.

**Supplementary Information:**

The online version contains supplementary material available at 10.1186/s13195-024-01667-6.

## Background

Sleep apnea is a common sleep-related breathing disorder characterized by repeated upper airway obstruction that results in apnea and hypoxemia during sleep. Sleep apnea is closely associated with several comorbid disorders, including cardiometabolic syndromes and neurodegenerative disorders [1, 2]. Recent studies have found that sleep apnea is associated with a higher risk of all-cause dementia and dementia of the Alzheimer type (DAT) [2–4]. However, few longitudinal studies on the relationship between sleep apnea and DAT in an Asian population have been published [4]. Given that Asians have a higher incidence of dementia and sleep apnea [4–6] and more deleterious effects of sleep apnea on comorbidities than Caucasians [6–8], it is necessary to reduce the knowledge gaps in the current understanding of the association between sleep apnea and dementia in Asians.

The prevalence of sleep apnea and dementia differs between men and women. The prevalence of sleep apnea in men is two to three times greater than that in women, whereas the prevalence of all-type dementia and DAT is 1.9 times greater in women than in men [2, 9]. Additionally, growing evidence shows that the effects of various risk factors on dementia are higher in women than in men [10, 11]. Therefore, the effects of sleep apnea on dementia may also differ depending on sex. However, the sex-specific relationship between sleep apnea and dementia remains unclear.

Obesity is the most important risk factor for sleep apnea [12, 13]. Moreover, there is sufficient evidence to suggest that obesity also puts an individual at risk of developing dementia [14]. However, previous studies failed to evaluate the different effects of sleep apnea on dementia according to the presence of obesity. We hypothesized that the deleterious effects of sleep apnea on dementia differ between non-obese and obese patients with sleep apnea.

Therefore, using data from the Korean National Health Insurance Service (KNHIS), we first investigated the association between sleep apnea and incident dementia (DAT and vascular dementia [VD]) in a Korean population. Second, we explored the effects of sleep apnea on dementia according to sex. Third, we sought to determine whether obesity affects the sex-specific relationship between sleep apnea and dementia after stratifying sleep apnea into non-obese and obese sleep apnea.

## Methods

### Data source

We used a customized dataset from the KNHIS, which includes more than 99% of Koreans (approximately 50 million) (http://nhiss.nhis.or.kr). The KNHIS database includes personal information; health insurance claim codes (procedures and prescriptions); diagnostic codes from the Korean Standard Classification of Diseases, 7th Revision (KCD-7) based on the International Classification of Diseases, 10th Revision (ICD-10); death records from the Korean National Statistical Office; and general medical examination data of each participant from 2002 to 2019. Body mass index (BMI) data were obtained from general health examinations in the KNHIS database.

This study was approved by the Institutional Review Board (IRB) of Korea University Guro Hospital and adhered to the principles of the Declaration of Helsinki (IRB No. 2022GR0280). Anonymous and de-identified data from the KNHIS were used for analysis and, therefore, the present study was exempted from obtaining informed consent.

### Study participants

Patients with sleep apnea (based on ICD-10 codes G47.30, G47.31, G47.32, or G47.38) aged ≥ 50 years between January 2002 and December 2015 were enrolled. A total of 50,964 eligible candidates were identified. We excluded 5,567 patients with a previous history of all cause of dementia or developing other forms of dementia (based on ICD-10 code F02, F03, G31 and the prescription of dementia medications) and 20,581 patients who did not have BMI measurement within 1 year before or after the diagnosis of sleep apnea. In addition, 271 underweight patients (BMI < 18.5 kg/m^2^) were excluded. Ultimately, 30,111 patients were included in the study. Patients with sleep apnea were classified according to the presence of obesity. Specifically, according to Asia-Pacific BMI criteria for obesity [15], patients with BMI < 25 kg/m^2^ were considered to have non-obese sleep apnea, whereas those with BMI ≥ 25 kg/m^2^ were classified as having obese sleep apnea.

The control group, which had not been diagnosed with sleep apnea between January 2002 and December 2015, was retrieved from the KNHIS database. Propensity score matching was performed to balance the age and sex distributions between the sleep apnea and control groups. Propensity scores were obtained using a multivariate logistic regression based on age and sex. A total of 121,528 individuals were matched with 30,382 patients with sleep apnea based on propensity scores using the 1:4 nearest-neighbor matching algorithm with a caliper of 0.2. After propensity score matching, 2,477 underweight individuals (BMI < 18.5 kg/m^2^) were excluded. Ultimately, 119,043 individuals were included in the control group in the present study.

### Definition of outcome and follow-up

The primary outcome of this study was the development of dementia (DAT and VD). DAT was defined according to ICD-10 code F00 or G30 and the prescription of dementia medications, including donepezil, rivastigmine, galantamine, and memantine. VD was defined according to ICD-10 code F01 and the prescription of dementia medications. Participants without dementia were considered to have completed the study at the end of the follow-up period. The participants were followed from the date of sleep apnea diagnosis to the date of the incident dementia or December 2022.

### Definition of covariates

Hypertension, diabetes, hyperlipidemia, ischemic stroke, hemorrhagic stroke, coronary heart disease, physical inactivity, heavy alcohol consumption, and a history of smoking were also considered. To improve the diagnostic accuracy of hypertension, diabetes, and hyperlipidemia, these conditions were defined according to both diagnostic codes and the prescription of medications. Specifically, the presence of hypertension was defined according to ICD-10 codes I10-15 and the prescription of antihypertensive medications. Diabetes was defined according to ICD-10 codes E8-14 and the prescription of antidiabetic medications. Hyperlipidemia was defined according to ICD-10 code E78 and the prescription of lipid-lowering medications. The presence of ischemic stroke was defined according to ICD-10 codes I63-66 and the prescription of antiplatelet or anticoagulation agents. Hemorrhagic stroke was defined according to the ICD-10 codes I60-62. Coronary heart disease was defined according to the ICD-10 codes I20-25 and the prescription of antiplatelet or anticoagulation agents. Physical inactivity was defined as physical exercise performed fewer than three times/week. Heavy alcohol drinking was defined as alcohol consumption at least three times per week. Smoking status was categorized into three groups: never-smokers, ex-smokers, and current smokers.

### Statistical analyses

Baseline characteristics are presented as mean ± standard deviation or median (interquartile range) and frequency (%). The characteristics of the sleep apnea and control groups were compared using independent t-tests and chi-square tests. All variables also met the proportional hazards assumption assessed by Schoenfeld residuals [16]. 

To check whether the impact of sleep apnea on the development of DAT or VD differs depending on sex, Cox proportional hazards regression analyses that included an interaction term between sex and sleep apnea were conducted using sleep apnea as a predictor and incident dementia (DAT or VD) as an outcome, after controlling for age, sex, obesity, hypertension, diabetes, hyperlipidemia, ischemic stroke, hemorrhagic stroke, coronary heart disease, physical inactivity, heavy alcohol consumption, and smoking status in the entire study population. Next, if interaction would be significant, we performed Cox proportional hazards regression with incident dementia (DAT or VD) as an outcome after controlling for the same potential confounders in each sex (women and men), while if interaction would not be significant, we performed Cox proportional hazards regression with the same model in the entire study population.

Finally, to determine whether obesity affects sex-specific relationships between sleep apnea and dementia, Cox proportional hazards regressions were conducted using the sleep apnea subtype (non-obese and obese sleep apnea) as a predictor and incident dementia (DAT or VD) as an outcome after controlling for age, sex, hypertension, diabetes, hyperlipidemia, ischemic stroke, hemorrhagic stroke, coronary heart disease, physical inactivity, heavy alcohol consumption, and smoking status for each sex (women and men) if interaction between sex and sleep apnea subtype (non-obese and obese sleep apnea) would be significant in the entire study population.

All reported *p*-values were two-sided and the significance level was set at 0.05. All analyses were performed using SAS version 9.4 (SAS Institute Inc., Cary, NC, USA).

## Results

### Clinical characteristics of the study participants

The demographic and clinical characteristics of the study participants are shown in Table 1. After propensity score matching, the mean age was not different between sleep apnea and control groups in both women (58.2 ± 5.9 and 58.2 ± 5.9 years) and men (57.9 ± 6.0 and 57.9 ± 6.0 years), respectively. However, there were differences in the frequency of obesity (women, 45.1% and 36.1%; men, 57.8% and 40.1%), hypertension (women, 42.3% and 31.3%; men, 48.1% and 34.9%), diabetes (women, 11.6% and 10.0%; men, 15.1% and 14.5%), hyperlipidemia (women, 52.1% and 36.8%; men, 48.7% and 31.0%), ischemic stroke (women, 3.7% and 1.8%; men, 5.4% and 2.7%), hemorrhagic stroke (women, 0.6% and 0.4%; men, 0.6% and 0.4%), and coronary heart disease (women, 6.3% and 3.5%; men, 11.5% and 5.8%) between sleep apnea and control groups, respectively, in both women and men.

**Table 1:** Baseline characteristics of participants

Variables	Women			Men		
	Sleep apnea (*n* = 11,319)	Control (*n* = 44,964)	*p*-value	Sleep apnea (*n* = 18,792)	Control (*n* = 74,087)	*p*-value
Age (years)	58.2 ± 5.9	58.2 ± 5.9	0.960	57.9 ± 6.0	57.9 ± 6.0	0.766
Obesity (n, %)	5,108 (45.13%)	16,221 (36.08%)	< 0.001	10,863 (57.81%)	29,698 (40.09%)	< 0.001
Hypertension (n, %)	4,783 (42.26%)	14,086 (31.33%)	< 0.001	9,035 (48.08%)	25,861(34.91%)	< 0.001
Diabetes (n, %)	1,308 (11.56%)	4,515 (10.04%)	< 0.001	2,834 (15.08%)	10,729 (14.48%)	0.038
Hyperlipidemia (n, %)	5,896 (52.09%)	16,542 (36.79%)	< 0.001	9,157 (48.73%)	22,939 (30.96%)	< 0.001
Ischemic stroke (n, %)	422 (3.73%)	821 (1.83%)	< 0.001	1,017 (5.41%)	2,000 (2.70%)	< 0.001
Hemorrhagic stroke (n, %)	68 (0.60%)	161 (0.36%)	< 0.001	117 (0.62%)	293 (0.40%)	< 0.001
CHD (n, %)	715 (6.32%)	1,566 (3.48%)	< 0.001	2,164 (11.52%)	4,268 (5.76%)	< 0.001
Systolic blood pressure	123.1 ± 14.8	123.6 ± 15.4	0.002	125.9 ± 13.8	126.7 ± 14.8	< 0.001
Diastolic blood pressure	76.0 ± 9.8	76.2 ± 9.9	0.135	78.8 ± 9.7	78.9 ± 10.0	< 0.001
Fasting glucose	100.5 ± 22.9	100.3 ± 25.4	0.570	105.2 ± 26.2	107.6 ± 32.0	< 0.001
Physical inactivity (n, %)	2,324 (20.57%)	8,878 (19.79%)	0.065	4,655 (24.83%)	16,718 (22.63%)	< 0.001
Heavy alcohol (n, %)	366 (3.24%)	1,538 (3.43%)	0.325	4,491 (23.94%)	20,710 (28.01%)	< 0.001
Smoking			< 0.001			< 0.001
Never smoker	10,727 (94.97%)	42,852 (95.54%)		6,067 (32.34%)	22,382 (30.27%)	
Ex-smoker	215 (1.90%)	592 (1.32%)		8,182 (43.61%)	25,064 (33.90%)	
Current smoker	353 (3.13%)	1,409 (3.14%)		4,512 (24.05%)	26,495 (35.83%)	
Median follow-up period	8.4 (7.0-9.7)	8.4 (7.1–9.7)		8.6 (7.1–9.8)	8.6 (7.1–9.8)	

Propensity score matching was performed to balance age and sex between the sleep apnea and control groups

Median follow-up period (years) was presented as median (IQR)

Abbreviation: CHD, coronary heart disease

### Cumulative incidence of DAT between sleep apnea and control groups

**Fig. 1:**
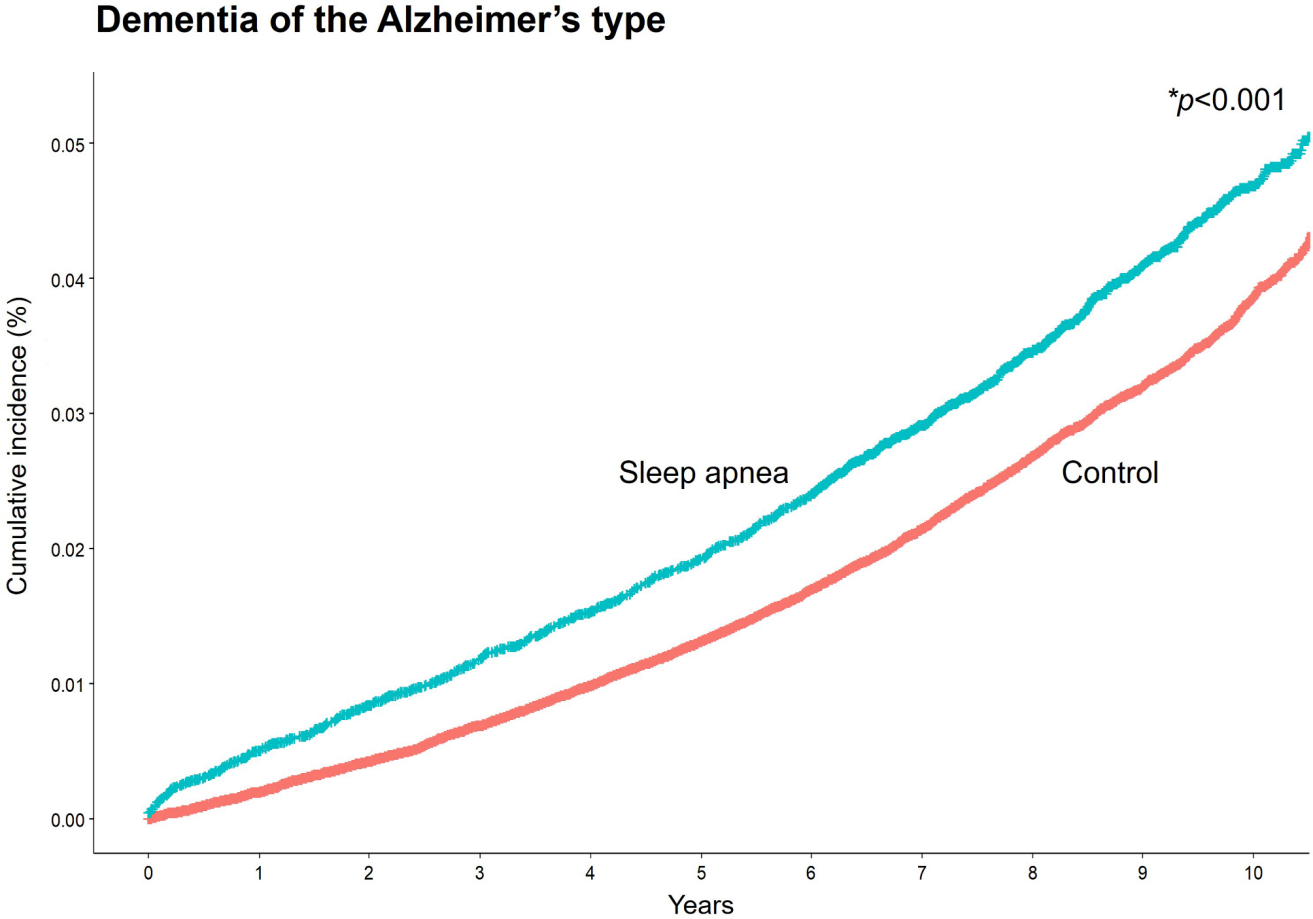
Cumulative incidence curves for DAT between sleep apnea and control groups *The cumulative incidence for DAT was compared with the log-rank test

Of the 30,111 patients with sleep apnea, 1,078 (3.6%) developed DAT, whereas 3,360 (2.8%) of the 119,051 participants in the control group experienced DAT (Table 2). As illustrated in Fig. 1, there was a higher cumulative incidence of overall DAT in the sleep apnea group than in the control group (hazard ratio [HR] = 1.26, 95% confidence interval [CI] 1.18–1.35, *p* < 0.001). Furthermore, after stratifying by sex, the cumulative incidence of overall DAT was higher in sleep apnea in the sleep apnea group than in the control group in both men (HR = 1.19, 95% CI 1.08–1.30, *p* < 0.001) and women (HR = 1.38, 95% CI 1.24–1.53, *p* < 0.001, supplementary Fig. 1).

**Table 2:** Incidence rate of dementia between sleep apnea and control groups

	Total		Women		Men	
	Sleep apnea	Control	Sleep apnea	Control	Sleep apnea	Control
DAT cases (n, %)	1,078 (3.58%)	3,360 (2.82%)	471 (4.16%)	1,372 (3.05%)	607 (3.23%)	1,988 (2.68%)
DAT incidence (per 100,000 person-years)	1.2	0.9	1.4	1.0	1.1	0.9
VD cases (n, %)	244 (0.81%)	771 (0.65%)	88 (0.78%)	278 (0.62%)	156 (0.83%)	493 (0.67%)
VD incidence (per 100,000 person-years)	0.3	0.2	0.3	0.2	0.3	0.2

Abbreviations: DAT, dementia of the Alzheimer type; VD, vascular dementia

### Impact of sleep apnea on the development of DAT in relation to sex

**Fig. 2:**
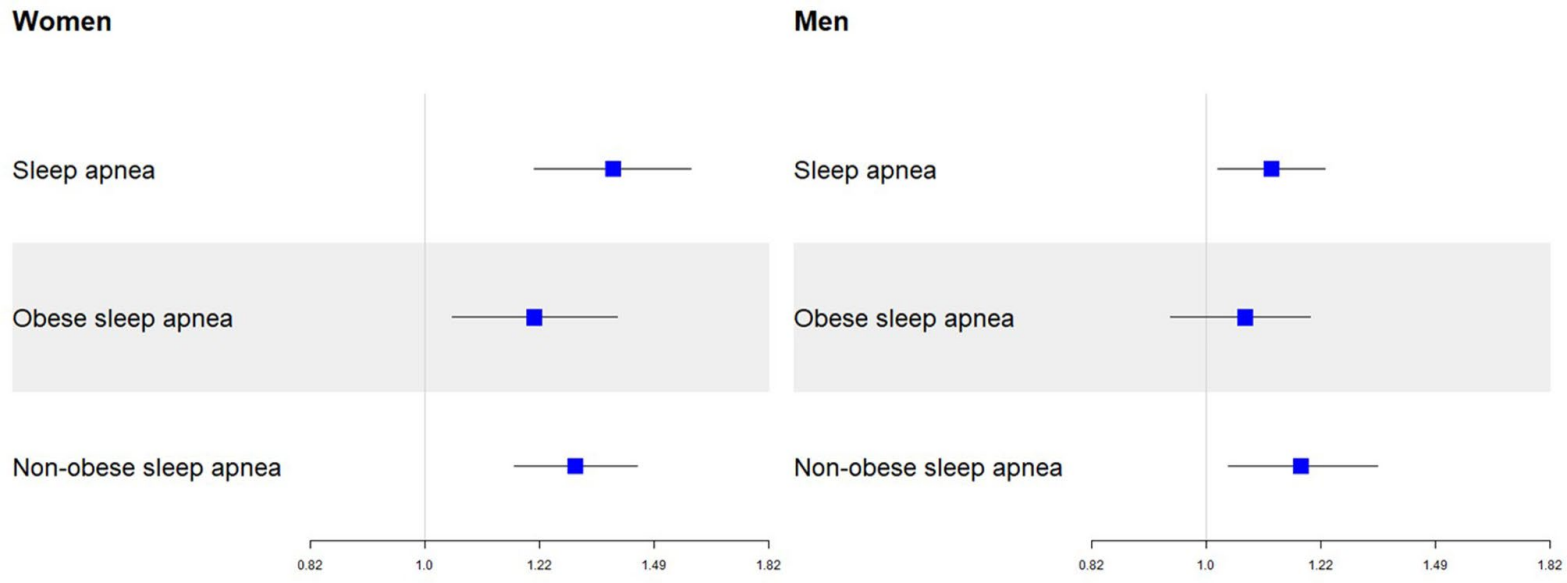
Association between sleep apnea and sleep apnea subtype with DAT in relation to sex Hazard ratios and 95% confidence intervals from the Cox proportional hazards regression models adjusted for age, sex, obesity, hypertension, diabetes, hyperlipidemia, ischemic stroke, hemorrhagic stroke, coronary heart disease, physical inactivity, heavy alcohol consumption, smoking status with sleep apnea (**A**), and sleep apnea subtype (**B**). Obesity was excluded as a covariate in the analysis with sleep apnea subtype as a predictor

There was an interaction between sleep apnea and sex with the development of DAT (*p* = 0.015 for sleep apnea×sex), suggesting that the effect of sleep apnea on development of DAT were different between sex. After stratification of sex, sleep apnea was associated with a higher risk of DAT in both women (HR = 1.30, 95% CI 1.16–1.44, *p* < 0.001) and men (HR = 1.13, 95% CI 1.03–1.24, *p* = 0.012; Table 3; Fig. 2). Especially, the effect of sleep apnea on the development of DAT was more prominent in women than in men (Table 3).

**Table 3:** Hazard ratio of sleep apnea for DAT

	Women		Men				
	HR (95% CI)*	*p*	HR (95% CI)*		*p*		*p* for interaction by sex**
Sleep apnea	1.30 (1.16–1.44)	< 0.001	1.13 (1.03–1.24)		0.012		0.015
Sleep apnea subtype							
Obese sleep apnea	1.39 (1.21–1.58)	< 0.001	1.06 (0.94–1.20)		0.329		0.002
	Entire study population						
	HR (95% CI)*			*p*		*p* for interaction by sex**	
Non-obese sleep apnea	1.18 (1.07–1.30)			< 0.001		0.667	

*Adjusted HR for DAT was obtained using Cox proportional hazards regression with sleep apnea or sleep apnea subtype as a predictor after controlling for age, sex, obesity, hypertension, diabetes, hyperlipidemia, ischemic stroke, hemorrhagic stroke, coronary heart disease, physical inactivity, heavy alcohol consumption, and smoking status in each sex. Obesity was excluded as a covariate in the analysis with sleep apnea subtype as a predictor

**p for interaction was estimated by the Cox proportional hazards regressions including sleep apnea or sleep apnea subtype and sex together as main effects and sex*sleep apnea or sex*sleep apnea subtype as an interaction effect after controlling for the same potential confounders in the entire study population

Abbreviations: CI, confidence interval; DAT, dementia of the Alzheimer type; HR, hazard ratio

### Impact of sleep apnea subtype (obese versus non-obese) on the development of DAT

Sleep apnea was stratified into obese sleep apnea and non-obese sleep apnea. There was an interaction between obese sleep apnea and sex with the development of DAT (*p* = 0.002 for obese sleep apnea×sex), suggesting that the effect of sleep apnea on development of DAT was different between sex. In women, obese sleep apnea (HR = 1.39, 95% CI 1.21–1.58, *p* < 0.001) increased the risk of DAT, whereas in men, obese sleep apnea (HR = 1.06, 95% CI 0.94–1.20, *p* = 0.329) did not increase the risk of DAT (Table 3).

However, there was no interaction between non-obese sleep apnea and sex with the development of DAT (*p* = 0.667 for non-obese sleep apnea×sex). Non-obese sleep apnea was associated with a higher risk of DAT in the entire study population (HR = 1.18, 95% CI 1.07–1.30, *p* = 0.001; Table 3).

### Impact of sleep apnea on the development of VD

There was no interaction between sleep apnea and sex with the development of VD (*p* = 0.874 for sleep apnea×sex). In the entire study population, sleep apnea (HR = 1.08, 95% CI 0.93–1.25, *p* = 0.305) was not associated with incident VD (Table 4). Neither obese (HR = 1.13, 95% CI 0.92–1.39, *p* = 0.233) nor non-obese sleep apnea (HR = 1.03, 95% CI 0.83–1.27, *p* = 0.792) was associated with VD (Table 4).

**Table 4:** Hazard ratio of sleep apnea for VD

	HR (95% CI)*	*p*	*p* for interaction by sex**
Sleep apnea	1.08 (0.93–1.25)	0.305	0.874
Sleep apnea subtype			
Obese sleep apnea	1.13 (0.92–1.39)	0.233	0.304
Non-obese sleep apnea	1.03 (0.83–1.27)	0.792	0.387

*Adjusted HR for VD was obtained using Cox proportional hazards regression with sleep apnea or sleep apnea subtype as a predictor after controlling for age, sex, obesity, hypertension, diabetes, hyperlipidemia, ischemic stroke, hemorrhagic stroke, coronary heart disease, physical inactivity, heavy alcohol consumption, and smoking status in the entire study population. Obesity was excluded as a covariate in the analysis with sleep apnea subtype as a predictor

**p for interaction was estimated by the Cox proportional hazards regressions including sleep apnea or sleep apnea subtype and sex together as main effects and sex*sleep apnea or sex*sleep apnea subtype as an interaction effect after controlling for the same potential confounders in the entire study population

CI, confidence interval; HR, hazard ratio; VD, vascular dementia

Abbreviation: DAT, dementia of the Alzheimer type

Abbreviation: DAT, dementia of the Alzheimer type

## Discussion

In this long-term follow-up of large cohort, we investigated the long-term effects of sleep apnea on incident dementia in non-obese and obese individuals. The major findings of this study are as follows. First, sleep apnea was predictive of DAT development. Second, the adverse effects of sleep apnea on DAT were more prominent in women than in men. Third, the adverse effects of obese sleep apnea on DAT were more pronounced in women than in men. In contrast, there were no differences in the effects of non-obese sleep apnea on DAT between women and men. Overall, the evidence suggests that obesity affected the sex-specific relationship between sleep apnea and DAT. Taken together, our findings suggest that sleep apnea exerted different effects on DAT development depending on sex and obesity. Therefore, sex-specific prevention strategies for sleep apnea and obesity may be necessary to prevent the development of DAT.

**Fig. 3:**
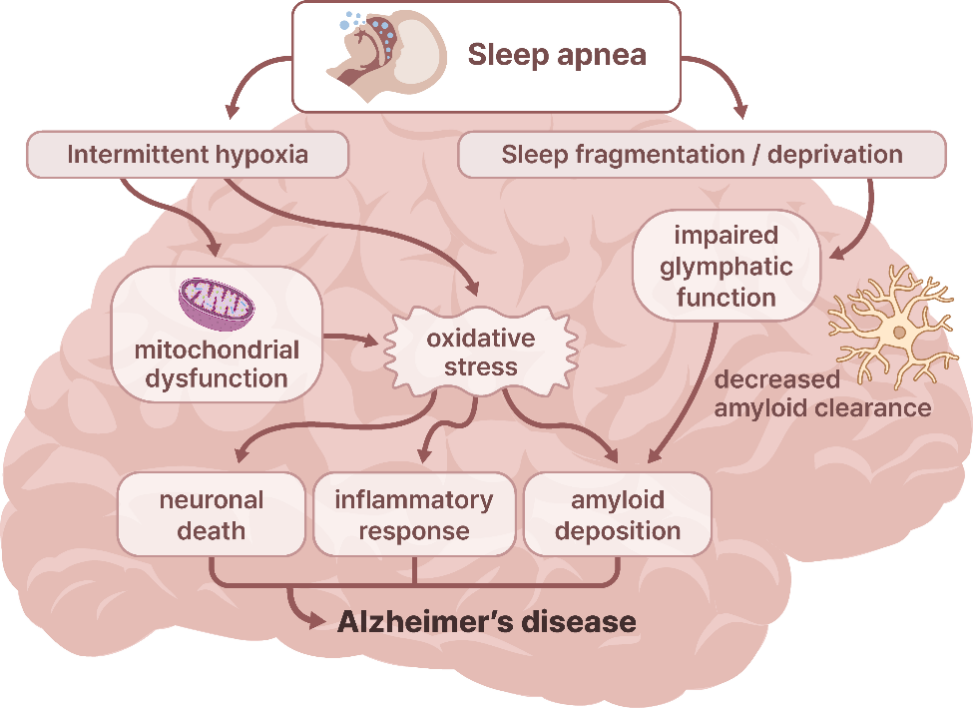
Proposed interplays between alzheimer’s disease and pathologic process caused by sleep apnea Sleep apnea causes oxidative stress directly via intermittent hypoxia and mitochondrial dysfunction, which further might lead to tissue damage, inflammation, amyloid deposition and neuronal death. Sleep deprivation is closely related to impaired glymphatic function leading to decreased clearance of amyloid in the brain

Our finding of an association between sleep apnea and DAT was consistent with those of previous studies based on multiethnic patients [3, 4]. Considering the differences in the effects of sleep apnea on other clinical complications, including cardiometabolic syndromes, between Caucasians and Asians [6–8], our findings should be emphasized to reduce the knowledge gaps in the current understanding of the association between sleep apnea and DAT in different ethnic populations. Although the mechanisms underlying this finding are not fully understood, intermittent hypoxia may mediate the relationship between sleep apnea and DAT. Several possible mechanisms include increased oxidative stress, systemic inflammation, mitochondrial dysfunction, cerebrovascular damage, metabolic dysregulation and blood-brain barrier hyperpermeability due to recurrent hypoxia, which may have detrimental effects on cognitive function [17, 18]. Especially, as illustrated in Fig. 3, sleep apnea causes oxidative stress directly via intermittent hypoxia and mitochondrial dysfunction, which in turn leads to tissue damage, inflammation, amyloid-β deposition and neuronal death involved in the pathophysiology of DAT [18, 19]. Oxidative stress-induced several by-products were also related with impaired cognition in various cognitive domains [18]. Antioxidants can decrease apoptosis in rat models of intermittent hypoxia, which suggests that treatment of sleep apnea can reduce inflammatory biomarkers associated with DAT [2, 20]. Other molecular mechanisms responsible for the cognitive deficits seen in sleep apnea include high levels of glutamate, which provoke excitotoxicity of the hippocampal neurons, and downregulation of hippocampal brain-derived neurotrophic factor [21]. Cerebrovascular damage may be another important mediator of the relationship between sleep apnea and DAT. Disruption of cerebral microcirculation, caused by sleep apnea, is a well-known pathogenesis of DAT [22]. In fact, previous studies have found that DAT patients with sleep apnea have an impairment in cerebrovascular disease markers closely associated with poor cognitive performance [23]. Neuroimaging studies have also found that sleep apnea reduces blood oxygen saturation, which causes brain atrophy mainly in the hippocampus, amygdala, frontal areas, and other functionally related regions with reduced cognitive flexibility [21, 24]. Sleep deprivation and sleep fragmentation are also responsible for the association between sleep apnea and DAT (Fig. 3). Especially, glymphatic dysfunction may mediate the association between sleep deprivation and DAT. Growing experimental evidence in rodent models has shown that glymphatic function, associated with clearance of metabolic waste, is activated during slow-wave sleep [25–27]. In this regards, sleep deprivation might cause a decreased glymphatic clearance, resulting in amyloid-β deposition [28]. Recently, the deleterious effect of sleep deprivation on impaired glymphatic function was found in humans [29], which in turn leads to neurodegeneration and cognitive decline [30]. Furthermore, several human studies have also found that sleep deprivation is closely associated with amyloid-β deposition in the brain [31–34]. These findings suggest that patients with sleep apnea are more susceptible to DAT.

Our major finding was that the adverse effects of sleep apnea on DAT were more prominent in women than in men. Furthermore, these differences were attributed to the effects of obese sleep apnea rather than non-obese sleep apnea. Several previous studies have investigated the moderating effects of sex on the relationship between sleep apnea and cognitive decline [35–38]. The observed sex-specific effect is consistent with the findings of two studies [36, 39], while contrasts with those of other studies [37, 38, 40]. Possible explanations for these discrepancy included the difference in study participants, design, and outcome. This sex-specific effect was strongly observed in middle-aged patient with sleep apnea (45 ~ 60 years) in previous studies. Our study included the middle-aged patients, which is why the sex-specific effect was evident. Furthermore, most studies used cognitive function tests as outcomes and, there a few studies exploring the development of DAT. Although the reason why female patients with sleep apnea are more vulnerable to DAT is not fully understood, our findings may be explained by the complex effects of biological and socioeconomic differences [41]. Several neuroimaging studies regarding sex-specific brain changes in patients with sleep apnea have shown white matter alterations [42], cortical thinning [43], and unilateral volume changes in the hippocampus [44] in women. Additionally, previous studies have found that sleep apnea and related intermittent hypoxia exert worse effects on the development of hypertension, cerebrovascular disease, and heart failure through endothelial dysfunction and a greater heart rate response and sympathetic nerve activity in women than in men. The differential effects of sleep apnea on several brain structures and systemic conditions have been attributed to differences in sex hormones, which are biological factors. In premenopausal women, estrogen has protective roles against the deleterious consequences of sleep apnea and intermittent hypoxia through increasing antioxidant activity and maintaining adequate muscle contracture of the upper airways [45, 46]. Additionally, progesterone is a respiratory stimulant which could reduce the frequency of apneas or airway obstruction during sleep [47]. However, women uniquely experience a menopause transition, which might increase the occurrence of sleep apnea and related complications via decreased estrogen and progesterone [48]. The loss of ovarian hormones following menopause could increase the development of sleep disorders and thus could lead to cognitive decline and dementia in women [49]. In fact, previous studies have found that the association between sleep apnea and poor cognitive performance is the strongest in perimenopausal women [36, 39, 50]. These results may suggest that estrogen decline is important in link between sleep apnea and DAT. Regarding socioeconomic factors, several studies have shown that women tend to maintain lifestyles that are more favorable for brain health, with overall lower drinking and smoking rates than men [51–54]. Therefore, our findings might also be related to differences in stress, alcohol consumption, smoking, and dietary habits according to sex, given that the adverse effect of the other risk factors including a poor lifestyle was so high that it eclipsed the effect of sleep apnea in men.

Our study highlights that obese women with sleep apnea are the most vulnerable to DAT. As BMI is positively correlated with oxygen desaturation severity, the impact of sleep apnea may be greater in obese patients than in non-obese patients due to exacerbation of oxygen desaturation during apnea events [55]. Interestingly, our findings showed an effect of sex differences in obese sleep apnea on DAT. Women have a unique sleep characteristics including shorter apneas, lower arousal threshold, and greater desaturation per arousal [56]. Thus, women may be more susceptible to severe oxygen desaturation during sleep, which could explain our finding that obesity and sleep apnea had a synergic effect on development of DAT in only women. Another reason for the vulnerability in women may include the shift of fat deposition from the subcutaneous to the visceral depot in menopausal women, which predisposes women to some cardiometabolic syndromes associated with obesity [57]. Moreover, obesity is more harmful in women than in men with respect to the risk of dementia, as proven by numerous clinical studies [58]. However, our findings suggest that sleep apnea in obese men is not related to DAT. As BMI is based on non-specific measures of height and weight, it may not reflect the correct volume of total body fat and muscle mass [59]. For an equivalent BMI, men have higher muscle mass than women, whereas women have greater amounts of total body fat than men [60]. Thus, it is possible that men with increased BMI may include metabolically healthy obese individuals with an increased muscle mass rather than in increased fat mass. Furthermore, a previous study found that only obese women were prone to develop DAT, whereas obese men were not [61]. In fact, increased BMI is related to the elevation of inflammatory proteins only in women, including C-reactive protein and interleukin-6, which can cause rapid cognitive decline due to an active inflammatory condition [62]. However, there has been very little research on obesity and sleep apnea linked to dementia directly, and there is no consistent evidence for the role of obesity in the association between sleep apnea and DAT.

Our findings that non-obese sleep apnea affects DAT in both women and men were contradictory to the relationship between obese sleep apnea and DAT, which might be attributed to different proportion of sleep apnea subtypes between obese and non-obese sleep apnea [63]. Non-obese sleep apnea is composed of a higher proportion of central or mixed sleep apnea than obese sleep apnea [63]. Central or mixed sleep apnea was closely associated with neurodegeneration, which directly affects the development of DAT without mediation of oxygen desaturation [64]. The patho-mechanism seems to be different depending on sleep apnea subtypes. However, further study is necessary to identify the exact mechanism and confirm this hypothesis.

We also found that sleep apnea was not associated with VD. Few studies have investigated the impact of sleep apnea on VD, although sleep apnea has been demonstrated to be an important risk factor for various cardiometabolic syndromes and cerebrovascular diseases. Our finding is consistent with the results of previous studies [65, 66]. These negative results might be explained by the fact these studies controlled for cardiometabolic risk factors, which might be mediators rather than confounders of the relationship between sleep apnea and VD.

### Limitations

The strengths of this study include the large sample size in the sleep apnea and control groups, well-balanced clinical demographics between the two groups, and long follow-up duration based on a nationwide cohort. However, our study has several limitations that should be addressed. First, the discordance between the diagnoses of sleep apnea and DAT in clinical practice and those recorded in the KNHIS (claims data) may lead to inaccurate results. Second, because the present study was performed using data that were not originally aimed at studying sleep disorders and neurodegenerative diseases, we could not assess the subjects’ sleep apnea severity, status of sleep apnea treatment, exact performance on cognitive function tests, baseline cognitive status, degree of depression, years of education, or occupation. Third, we did not consider information on exposure time and changes in the status of sleep apnea. Fourth, we excluded the underweight individuals, which could affect the external validity. However, these issues might be mitigated by the fact that underweight individuals have a lower prevalence of sleep apnea [67]. In fact, the proportion of being underweight in sleep apnea group was very low (0.89%) in the present study. Fifth, because we used Asia-Pacific BMI criteria for obese participants, caution should be exercised when generalizing our findings to other ethnicities. Sixth, the generalizability of this study to community-based populations is limited given that the cohort was recruited from general health examinations setting in Korea, which results in the enrollment of a more “health-seeking” population. Seventh, because we defined VD according to ICD-10 code and the prescription of dementia medications, VD incidence might be underestimated. Finally, although the impacts of sleep apnea on DAT were identified, the mechanism for our findings could not be confirmed. Thus, further study is needed to identify the exact patho-mechanism. Nevertheless, our study is noteworthy because it included a larger number of patients with sleep apnea and a longer follow-up period than previous studies and was based on an Asian population.

## Conclusions

In the present study, we highlighted sex differences in the adverse effects of sleep apnea on DAT. Furthermore, our findings suggest that sex-specific prevention strategies for controlling sleep apnea are necessary to prevent DAT.

## Electronic supplementary material

Below is the link to the electronic supplementary material.

Supplementary Material 1

## Data Availability

The Korean NHIS database is confidential, and has been approved for use by researchers who meet the criteria for access through the Korea National Health Insurance Sharing Service (NHISS) Institutional Data Access Committee (https://nhiss.nhis.or.kr/bd/ay/bdaya001iv.do). If data were requested for additional analysis, the corresponding author would deliberately consider offering after passing the review process of the Korea NHISS Institutional Data Access Committee and after payment of the data access fee charged to the requester.
