# Supplementary material for: Impact of sleep apnea on alzheimer’s disease in relation to sex: an 8-year longitudinal follow-up study of a nationwide cohort
Source: Alzheimers Res Ther. 2025 Mar 20;17:65. doi: 10.1186/s13195-024-01667-6 (PMC11924715; doi:10.1186/s13195-024-01667-6)
Supplement: Supplementary file 1 — Supplementary Material 1 [file 13195_2024_1667_MOESM1_ESM.docx]

**
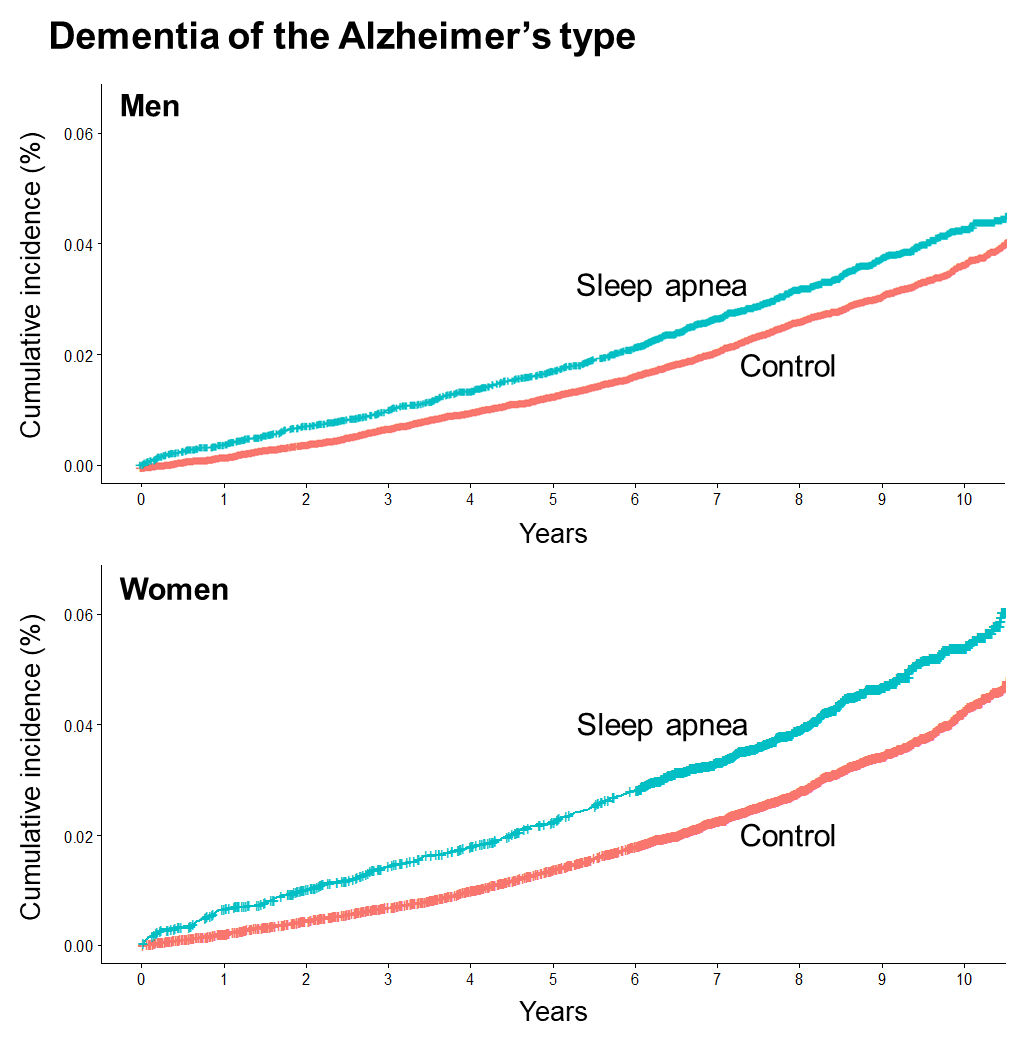
**

**Supplementary Figure 1.** Cumulative incidence curves of DAT between sleep apnea and control groups in men and women

*The cumulative incidence for DAT was compared with the log-rank test in each sex.

Abbreviation: DAT, dementia of the Alzheimer type.
